# The impact of repetitive navigated transcranial magnetic stimulation coil positioning and stimulation parameters on human language function

**DOI:** 10.1186/s40001-015-0138-0

**Published:** 2015-04-01

**Authors:** Nico Sollmann, Sebastian Ille, Thomas Obermueller, Chiara Negwer, Florian Ringel, Bernhard Meyer, Sandro M Krieg

**Affiliations:** Department of Neurosurgery, Klinikum rechts der Isar, Technische Universität München, Ismaninger Str. 22, 81675 Munich, Germany; TUM-Neuroimaging Center, Klinikum rechts der Isar, Technische Universität München, Ismaninger Str. 22, 81675 Munich, Germany

**Keywords:** Transcranial magnetic stimulation, Navigated brain stimulation, Cortical mapping, Language, Stimulation protocol, Object naming

## Abstract

**Background:**

Repetitive navigated transcranial magnetic stimulation (rTMS) in combination with object naming is able to elicit naming errors by stimulating language-related brain regions. However, stimulation results mainly depend on coil positioning and stimulation parameters, which have not been investigated since the implementation of neuronavigation to transcranial magnetic stimulation. Therefore, the following three parameters were systematically examined in the present study: coil angulation, stimulation frequency, and stimulation intensity.

**Methods:**

Five healthy, right-handed subjects underwent rTMS language mapping of Broca’s as well as Wernicke’s areas of the left hemisphere. During mapping sessions, coil angulation was changed clockwise in 45° steps, and the stimulation frequency and intensity were varied within a considerably wide range. For angulation, the anterior-posterior (ap) coil orientation was used as reference position.

**Results:**

An angulation of 90° to ap coil orientation led to the highest rate of naming errors within Broca’s area, whereas an inhomogeneous distribution of angulations was observed during stimulation of Wernicke’s area. Therefore, ap coil orientation, which is regarded as standard in rTMS language mapping, could not be approved as the optimal position. With regard to stimulation parameters, 20 Hz and 120% of the resting motor threshold (RMT) were defined as optimal.

**Conclusions:**

Coil angulation, stimulation frequency, and stimulation intensity have significant impacts on language impairment during rTMS mapping. The variation of only one of these parameters already leads to a clearer disruption of language performance. Therefore, individually adapted stimulation protocols have to be determined prior to language mapping in order to improve mapping results.

## Background

Over the last years, transcranial magnetic stimulation (TMS) has been increasingly used for diagnostic and therapeutic purposes. In general, TMS is a non-invasive method that induces an electrical field, which indirectly and transiently excites or inhibits pyramidal neurons. Navigated transcranial magnetic stimulation (nTMS), which is a combination of a TMS unit and a neuronavigation system, features a simultaneous 3D tracking and visualization of cortical stimulation points [1]. With regard to its therapeutic options, it has already been used for the treatment of a variety of psychiatric and neurological disorders, especially depression [2-4], chronic tinnitus [5-7], and chronic pain [8-10]. Concerning diagnostic purposes, nTMS has started to play an important role for neurosurgical operation planning as it can also be used to map functionally relevant brain areas, which has surgical implications for resectable/non-resectable decision-making. Nowadays, as part of a multimodal setup, nTMS is primarily used for the preoperative mapping of motor- and language-related brain regions [11-16]. While established stimulation protocols are already available for nTMS motor mapping, there is no reliable and standardized stimulation protocol for the mapping of language-related brain areas by repetitive nTMS (rTMS). In general, rTMS language mapping results depend on a variety of different parameters, especially coil angulation, stimulation frequency, and stimulation intensity. Variation of only one of these parameters can already lead to a different TMS impact on language performance, which has been known since the publication of Epstein *et al*.’s examination of different stimulation settings [17]. Although it is one of the main approaches focused on the relationship between rTMS parameters and language impairment, this study does not provide a systematic examination of numerous coil angulations. However, the targeting of the stimulation coil is already known to be crucial, and small rotations can already alter rTMS language mapping results [18].

As further development and standardization of the rTMS language mapping procedure seems to be essential for the successful use of TMS technique, we systematically examined the effects of the three already mentioned parameters on language performance. Therefore, five healthy and purely right-handed subjects underwent language mapping of Broca’s and Wernicke’s areas of the left hemisphere through rTMS combined with an object naming task. Coil angulation, stimulation frequency, and stimulation intensity were varied, and the results of language disruption were evaluated.

## Methods

### Subjects

Five healthy subjects underwent rTMS language mapping of Broca’s and Wernicke’s areas of the left hemisphere. All volunteers indicated German as their mother tongue, and right-handedness was approved by the Edinburgh Handedness Inventory (EHI) in all subjects. Three volunteers were female, and two were male.

This study was conducted with the consent of the local ethics committee of Technische Universität München (registration number: 2793/10) and in accordance with the Declaration of Helsinki. Written informed consent was obtained from all volunteers prior to MR imaging.

### MRI data acquisition

Before rTMS language mapping, all subjects underwent magnetic resonance imaging (MRI). This was performed on a 3-T MRI scanner combined with an eight-channel phased array head coil (Achieva 3 T, Philips Medical Systems, Best, The Netherlands B.V.) without intravenous contrast administration. The protocol for MRI data acquisition was repeatedly used and described in other rTMS language mapping studies [16,19-21]. The 3D MRI dataset was transferred to the nTMS system using the DICOM standard.

### rTMS language mapping

#### Experimental setup

rTMS language mapping was performed with the same Nexstim eXimia NBS system, version 4.3, with a NexSpeech® module (Nexstim Oy, Helsinki, Finland) in all cases. In short, this system provides an electromagnetic stimulation coil in combination with a neuronavigation unit, which allows simultaneous 3D tracking of the coil and visualization of all stimulation sites [1]. During rTMS, the coil induces an electrical field, which is visualized over the individual 3D MRI reconstruction images of each volunteer’s brain, and the intracranial stimulation points are saved for later examination [1,22]. Our setup follows the protocol of previous studies on rTMS language mapping [12,16,18-21,23-25]. Only biphasic stimulation pulses were applied throughout the mappings.

#### Determination of the resting motor threshold

As a part of preparation for rTMS language mapping, all volunteers underwent the same procedure to determine the individual resting motor threshold (RMT). Therefore, motor evoked potentials (MEP) of the right abductor pollicis brevis (APB) muscle were measured by an electromyography (EMG) unit while stimulation impulses were applied over the left-hemispheric motor cortex as described in earlier reports [13,14,26]. The induced electrical field was oriented perpendicular to the left-hemispheric precentral gyrus that was stimulated for RMT determination [13,14,26].

#### Object naming and baseline testing

As a common and frequently used task for language function testing, object naming, which engages all three major language production functions (articulation, meaning, and form), was used for baseline testing and rTMS language mapping, as recently published [19-21,27].

During baseline testing, 131 colored photographs of familiar living and non-living objects were displayed on a screen in front of the volunteer at an inter-picture interval (IPI) of 2.5 s and without simultaneous stimulation. Every subject was instructed to name all objects in German as precisely and quickly as possible. Misnamed objects were immediately discarded from the object sequence. After the first baseline testing session, a second one with the stack of remaining images was performed in an analog way. The remaining objects, after second baseline testing, were used during the stimulation session. For later analysis, the baseline performances were digitally video recorded [19,24].

#### Language mapping procedure

**Figure 1:**
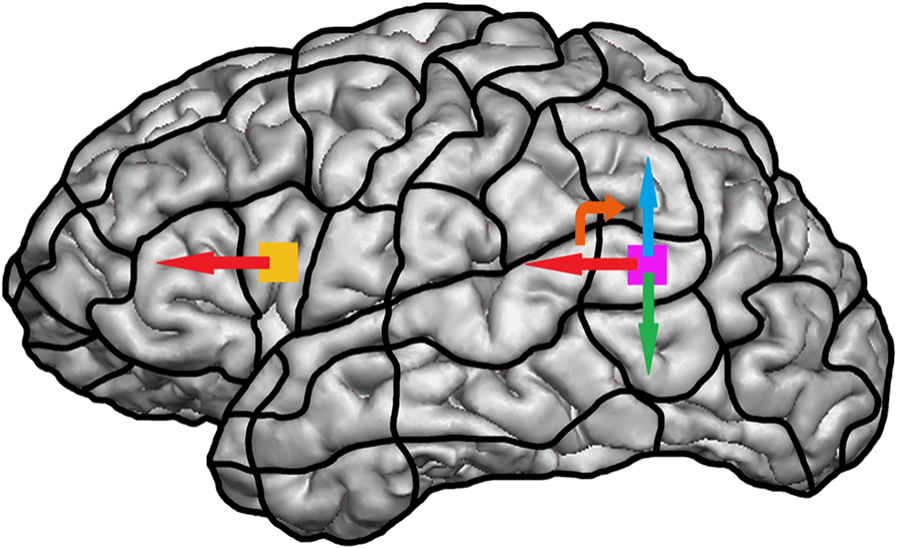
**Stimulation spots within Broca’s area (orange spot) and Wernicke’s area (purple spot), visualized on a parcellated brain.** The straight arrows symbolize ap (red arrows, reference position), 90° (blue arrow), and 270° (green arrow) coil orientation. Moreover, the orange, curved arrow indicates the direction of coil rotation.
  - Stimulation frequency: stimulation with 5, 7, 10, and 20 Hz/5 pulses in ap and optimal coil orientation (as found in Figure 2a); 10 stimulations per position and frequency (4 frequencies × 2 positions × 10 = 80 stimulations) with 100% RMT.

After determination of the individual RMT and baseline testing, language mapping was performed in order to examine the impact of three different parameters on language performance: coil angulation, stimulation frequency, and stimulation intensity. Therefore, all volunteers underwent rTMS of Broca’s and Wernicke’s areas of the left hemisphere based on the following protocol:

1. A train of rTMS bursts was administered to Broca’s and Wernicke’s areas of the left hemisphere in order to identify one cortical site with clear and reproducible no-response errors each, based on the volunteer’s and examiner’s impressions and supported by video analysis. For determination of optimal cortical spots, mapping was performed with anterior-posterior (ap) coil orientation, 5 Hz/5 pulses (duration: 1.0 s), and 100% of the individual RMT. These values are most frequently used as starting parameters in rTMS-based language investigations [19,21,28-30].
2. At these two sites with reproducible language impairments, coil angulation and stimulation frequency and intensity were varied according to the following chronological order:
  - Coil angulation (Figure 1): variation in steps of 45° (beginning with ap coil orientation); 10 stimulations per position (8 positions = 80 stimulations) with 5 Hz/5 pulses (duration: 1.0 s) and 100% RMT.

**Figure 2:**
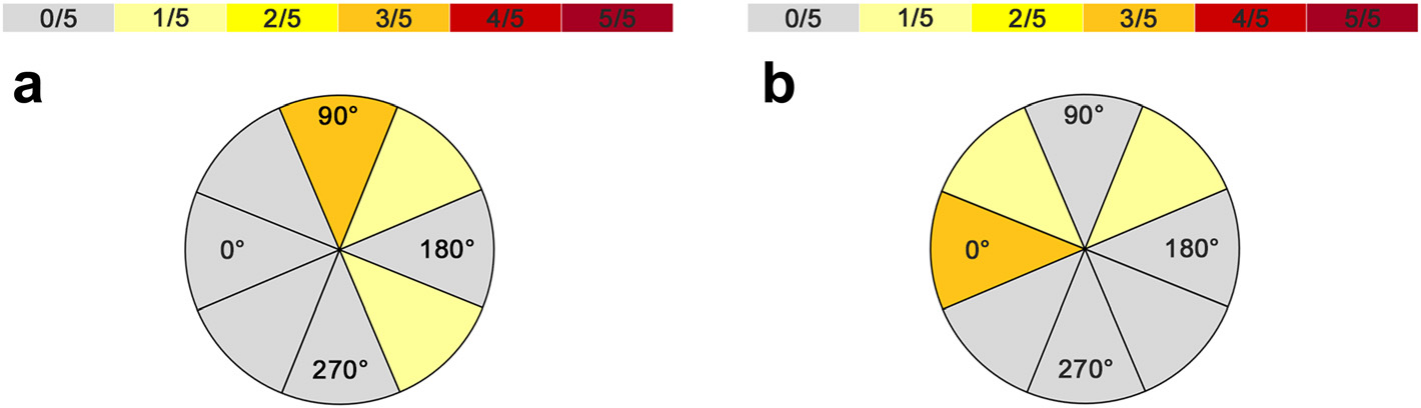
**The circle diagram visualizes the distribution of optimal coil angulations within Broca’s area in relation to ap coil orientation (a) as well as in relation to the reference gyrus (b), and the number of subjects who were prone to most of the errors at these angulations.**
  - Stimulation intensity (% RMT): stimulation with 100%, 80%, and 120% RMT in ap (with 5 Hz/5 pulses) and optimal coil orientation (with the optimal frequency as found in Figure 2b); 10 stimulations per position and intensity (3 intensities × 2 positions × 10 = 60 stimulations).

Broca’s and Wernicke’s areas were anatomically identified on each volunteer’s MRI, meaning that during step 1 of our stimulation protocol, rTMS bursts were administered to the triangular and opercular parts of the inferior frontal gyrus (Broca’s area) and to the posterior superior temporal gyrus and angular gyrus (Wernicke’s area) to detect cortical sites with clear and reproducible no-response errors, as described above. Basically, both areas have shown to be especially prone to naming errors during rTMS in recent trials [24,25,31], which made them appropriate for systematic assessment of the three aforementioned parameters.

According to the stimulation protocol, a total number of 2 × (80 + 80 + 60) = 440 stimulation trains were applied to each volunteer’s cortex. The set of objects named correctly during baseline testing was displayed time-locked to a train of rTMS pulses. All objects were presented in a randomized endless loop mode with an IPI of 2.5 s, and rTMS pulse trains were started 0 ms after the picture presentation onset. The coil was placed tangential to the skull in order to achieve maximum field induction [17,24,32]. For investigating the impact of coil orientation on language function, the angulation of the magnetic coil was changed during the IPI after 10 stimulations. We defined the ap orientation as the starting position, which means that the corresponding electrical field generated by the magnetic coil is angulated horizontally with respect to a line between external acoustic meatus and nasion (Figure 1). This position is commonly regarded as the standard position for rTMS-based language mappings [17,19,24,33].

By causing a virtual functional lesion, rTMS is able to identify cortical regions causally related to language functions [34-36]. For an objective and detailed analysis, all individual mapping session performances were digitally video recorded [19,24].

To evaluate discomfort during stimulation for safety and interpretability of the disrupted naming performance, each volunteer was asked to rate perceived pain according to the visual analog scale (VAS) from 0 (no pain) to 10 (maximum pain) divided into temporal muscle pain and pain during rTMS at convexity.

#### Data analysis

All language mapping data were examined based on the videos after the investigation as described previously [16,19-21,24]. Any rTMS-induced disturbance of language was compared with the corresponding baseline performance, and clear naming errors were counted [37].

Moreover, optimal mapping parameters and optimal coil angulation were defined as the settings that lead to the highest number of language disturbances in total. Coil angulation was measured with ap coil orientation or gyrus as references (Figure 1). For these optimal settings, electrical field strength was evaluated at Broca’s and Wernicke’s area stimulation sites.

A chi-square test or Mann–Whitney-Wilcoxon test was performed to test the distribution of attributes. Results are presented as mean ± standard deviation (SD) or individual scores. For interpretation, *P* values were calculated, and *P* < 0.05 was considered significant.

## Results

### Subject-related characteristics

Relevant subject-related characteristics, including age, handedness score based on the EHI, number of correctly named baseline objects, individual RMT, and pain score, according to the VAS are provided in Table 1.

**Table 1:** **Subject-related characteristics**

**Subject**		**#1**	**#2**	**#3**	**#4**	**#5**	**Mean ± SD**	***P*** **values**
Age (years)		24	26	29	26	23	25.6 ± 2.3	-
Handedness (EHI)		100	57	100	100	60	83.4 ± 22.8	
Correctly named baseline objects (out of 131)		111	115	121	113	105	113.0 ± 5.8	
RMT (% output)		40	28	42	41	35	37.2 ± 5.8	
Pain (VAS)	Broca	7	5	2	4	5	4.6 ± 1.8	<0.0001
	Wernicke	2	1	1	2	3	1.8 ± 0.8	

### rTMS language mapping

**Figure 3:**
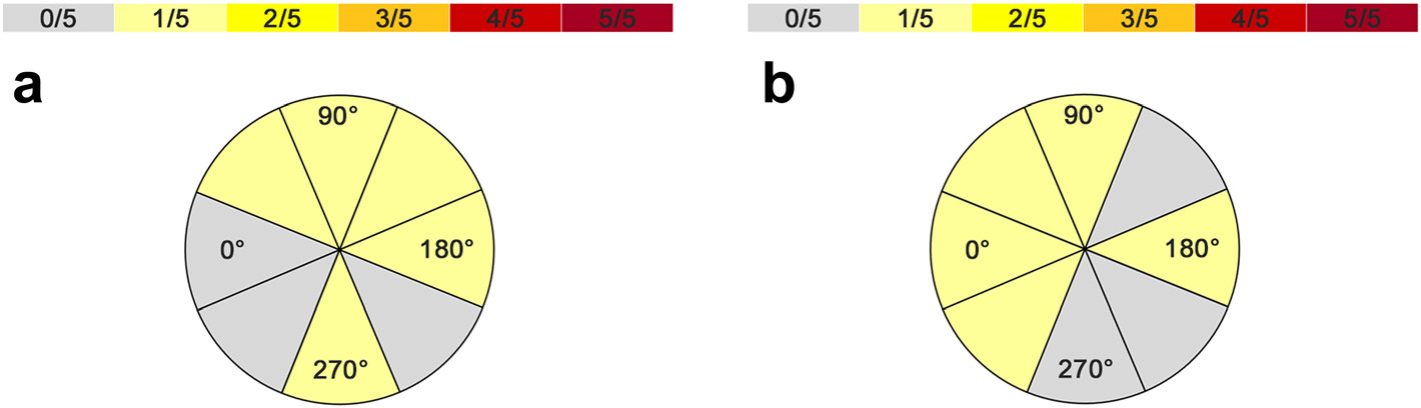
**The circle diagram visualizes the distribution of optimal coil angulations within Wernicke’s area in relation to ap coil orientation (a) as well as in relation to the reference gyrus (b), and the number of subjects who were prone to most of the errors at these angulations.**

Ap coil orientation was defined as the optimal position in no case (Tables 2 and 3, Figures 2a,b and 3a,b). Higher stimulation frequencies and higher stimulation intensities led to an increased number of naming errors with regard to Broca’s and Wernicke’s areas in general (Tables 2, 4, and 5). In this context, within Broca’s area, an angulation of 90° to ap coil orientation led to the highest number of naming errors in three out of five cases (Tables 2 and 3, Figure 2a). Concerning coil orientation in relation to the reference gyrus, 0° was defined as optimal in three cases during rTMS language mapping of Broca’s area (Tables 2 and 3, Figure 2b). For Wernicke’s area, an inhomogeneous distribution of coil angulations relative to ap coil orientation and to the reference gyrus was observed (Tables 2 and 3, Figure 3a,b). Furthermore, 20 Hz was defined as the optimal frequency in two out of five cases within Broca’s area and in three out of five cases within Wernicke’s area (Tables 2 and 4). During stimulation of Broca’s and Wernicke’s areas, an intensity of 120% RMT was defined as optimal in three out of five cases, whereas 100% RMT led to the most language disturbances in two out of five cases (Tables 2 and 5).

**Table 2:** **Summary of stimulation parameters and coil angulations**

	**Broca**	**Wernicke**	***P*** **values**									
**Subject**	**#1**	**#2**	**#3**	**#4**	**#5**	**#1**	**#2**	**#3**	**#4**	**#5**	---	
Optimal coil angulation (in °)	To ap orientation	225	90	90	135	90	90	45	180	270	135	0.8288
	To reference gyrus	135	0	0	45	0	0	315	90	180	45	0.1945
Optimal stimulation frequency (in Hz)		10	10	20	20	7	20	20	20	7	10	0.7337
Optimal stimulation intensity (% RMT)		100	120	120	120	100	120	100	100	120	120	0.9025
Electrical field strength (in V/m)		85	120	95	100	70	95	75	105	90	75	0.7526

**Table 3:** **Distribution of naming errors in relation to coil angulations**

	**Broca**						**Wernicke**					
**Subject**	**#1**	**#2**	**#3**	**#4**	**#5**	**Totals**	**#1**	**#2**	**#3**	**#4**	**#5**	**Totals**
0°	5	3	9	6	8	31	3	2	6	3	8	22
45°	2	4	5	4	7	22	2	4	1	3	6	16
90°	5	7	10	6	9	37	6	3	2	4	5	20
135°	5	4	4	7	6	26	3	2	2	4	10	21
180°	3	1	3	6	8	21	4	2	7	2	8	23
225°	6	3	5	4	8	26	3	3	4	3	7	20
270°	4	3	7	4	7	25	3	1	4	5	7	20
315°	3	2	4	3	8	20	1	2	4	2	6	15
Optimal angulation (in °)	225	90	90	135	90	-	90	45	180	270	135	-

**Table 4:** **Distribution of naming errors in relation to stimulation frequencies**

	**Broca**						**Wernicke**					
**Subject**	**#1**	**#2**	**#3**	**#4**	**#5**	**Totals**	**#1**	**#2**	**#3**	**#4**	**#5**	**Totals**
5 Hz ap	2	3	6	3	9	23	3	3	3	2	8	19
5 Hz opt.	2	4	9	6	7	28	4	3	6	3	7	23
7 Hz ap	3	4	2	4	9	22	2	3	2	2	8	17
7 Hz opt.	4	3	7	6	10	30	4	1	5	6	8	24
10 Hz ap	2	3	9	3	10	27	3	4	6	4	9	26
10 Hz opt.	8	6	9	4	9	36	2	4	6	4	10	26
20 Hz ap	4	5	9	6	9	33	3	3	5	3	9	23
20 Hz opt.	4	5	10	7	9	35	5	5	8	5	9	32
Optimal frequency (in Hz)	10	10	20	20	7	-	20	20	20	7	10	-

**Table 5:** **Distribution of naming errors in relation to stimulation intensities**

	**Broca**						**Wernicke**					
**Subject**	**#1**	**#2**	**#3**	**#4**	**#5**	**Totals**	**#1**	**#2**	**#3**	**#4**	**#5**	**Totals**
100% RMT 5 Hz ap	3	2	7	3	7	22	3	2	7	4	7	23
120% RMT 5 Hz ap	4	3	10	5	7	29	3	3	7	5	10	28
80% RMT 5 Hz ap	2	2	3	3	6	16	3	2	2	3	5	15
100% RMT opt.	7	3	9	4	10	33	4	5	10	4	9	32
120% RMT opt.	5	4	10	5	8	32	5	2	8	6	10	31
80% RMT opt.	5	3	9	4	6	27	4	3	7	5	7	26
Optimal intensity (% RMT)	100	120	120	120	100	-	120	100	100	120	120	-

## Discussion

### Coil angulation

The most interesting finding seems to be the fact that ap coil orientation, which is regarded as the standard position in rTMS language mapping [17,19,24,33], was defined as the optimal position in no case (Tables 2 and 3, Figures 2a,b and 3a,b). With regard to Broca’s area, Epstein *et al*., who tried to define optimal and tolerable TMS parameters for safe cortical language mapping in a cohort of six individuals, came to the conclusion that strict horizontal alignment of the induced electrical field elicits the highest rate of no-response errors [17]. In their study, they performed TMS language mapping with coil angulations of 0° and 90°. Despite the fact that the authors only used two different angulations (rather than a larger range of orientations, as we did in our study), it is obvious that our results are not in concordance with their results at all. They also described pain as clearly more intensive during stimulation with 90° due to the potential direct stimulation of temporal muscle fibers [17] - an observation that cannot be approved by our data. Although our study results do not include explicit VAS scores for perceived pain during stimulation at each coil orientation, each subject was asked to rate the pain immediately after stimulation. None of the five volunteers enrolled in our investigation described one special angulation as more painful than another. rTMS of Broca’s area is not completely painless under any circumstances, but with regard to coil positioning, none of the eight angulations led to more or less discomfort compared to the others.

Up to now, the literature about the exact correlation between coil orientation in relation to the reference gyrus or ap direction and error evocation has been very rare and has never been systematically investigated. As has been mentioned, some authors have reported that variations of coil angulation led to different rTMS language mapping results in their studies [17,18], but an explanation of this observation is missing. Therefore, a reasonable, literature-based analysis of our results regarding coil orientation seems to be crucial. As one interpretation of our rTMS mapping results, it seems to be possible that different coil angulations interfere with different subcortical tracts, which can lead to an angulation-correlated grade of language impairment. In this context, angulation changes of an nTMS coil placed on the precentral gyrus for cortical motor mapping can lead to an increase in motor response latency out of the commonly known latency range of monosynaptic MEPs [38], meaning that nTMS presumably stimulated different and more distant motor-related structures in an angulation-dependent way. The same might principally be true for rTMS language mapping, meaning that different coil angulations could interfere with slightly different language-related tracts despite the spot of cortical stimulation stays the same. However, this hypothesis is not yet supported by the previous literature, and it remains questionable whether it can be justified in the light of future investigations.

Furthermore, *P* values calculated for the comparison of Broca’s *vs*. Wernicke’s optimal coil angulations in relation to the reference gyrus and ap orientation indicate no significance (Table 2). This finding shows that, at least in our study, there is no correlation between the optimal coil orientations at these two areas for rTMS language mapping, which means that the coil angulation at one of these spots cannot predict the other one’s optimal angulation. Therefore, coil angulations should be determined separately for Broca’s and Wernicke’s areas in order to improve the mapping results of the individual subject. However, the small sample size of this pilot study limits the result.

For rTMS language mapping of a single cortical spot, testing of different coil angulations in order to obtain optimal mapping results seems applicable. In contrast, a variation of coil orientation during stimulation of large areas or whole hemispheres is likely to become a demanding or even impossible task for the examiner, as he/she would have to change coil orientation and coil localization simultaneously within the IPI. Therefore, further investigations to detect single rTMS coil angulations that lead to optimal language impairment among most individuals are highly preferable. Because the cohort of our study is too small and optimal coil angulations for stimulation of Wernicke’s area were distributed inhomogeneously among all volunteers, a definite recommendation for optimal coil orientation for rTMS language mapping in general cannot be made yet. Nonetheless, since previous studies on non-navigated rTMS language mapping did not investigate or discuss the matter of coil orientation at all, the results of this first systematic trial can be seen as the base for further studies on this topic [17,24,33,39,40]. To also evaluate the influence of subcortical or cortico-cortical fiber tracts, a new trial will have to combine rTMS language mapping with diffusion tensor imaging fiber tracking.

### Stimulation frequency

In general, higher stimulation frequency was correlated with higher numbers of naming errors according to our stimulation results (Tables 2 and 4). When our stimulation frequency findings are compared to those of Epstein *et al*., results are not in concordance once again [17]. In their study, the authors used five different frequencies (2, 4, 8, 16, and 32 Hz) for rTMS language mapping [17]. Their results illustrated that slower repetition rates of TMS pulses led to clearer language impairment with special regard to no-response errors because stimulation with 4 and 8 Hz elicited speech arrests in six out of six subjects, whereas rTMS with 16 and 32 Hz only lead to speech arrests in two out of six subjects [17]. In our study, the highest examined stimulation intensity (20 Hz) was correlated with most of the language disturbances that were observed, whereas low frequencies (5 and 7 Hz), which were comparable to those used in the study of Epstein *et al*., only elicited clear naming errors occasionally [17]. In a different study of our research group, a study that used 5 and 7 Hz for triplicate language mapping among 10 healthy volunteers, stimulation with higher frequency (7 Hz) again elicited more language disturbances than low-frequency mapping [21]. Moreover, when not only focusing on one modality for the identification of human language-related brain areas, at electrocorticography (ECoG), for example, a relatively high frequency is usually considered optimum for language localization, and this can lead to the assumption that higher frequencies would be more effective for rTMS language mapping as well [17,41].

In a study of Pascual-Leone *et al*., rTMS language mapping was performed with frequencies of 8, 16, and 25 Hz in six patients, and 8 and 16 Hz led to the greatest number of no-response errors in three patients each [39]. Therefore, this study seems to partially correspond with Epstein *et al*.’s study and our present study because half of the individuals were prone to naming errors during low frequency and half of the subjects were prone to naming errors during high-frequency rTMS [17,39]. In a study published this year, authors Rogic *et al*. performed language mapping with high-frequency rTMS (12 Hz) and found that language disruptions were successfully produced in all subjects [25]. Moreover, further literature review also tends to indicate that language impairment is more likely to be caused by high frequency than by low-frequency stimulation [40,42].

In addition, some authors reported that rTMS using high frequency is related to a higher rate of unclear language disturbances due to dysarthria or intolerable pain [17,39]. We came to the same results regarding these two aspects after evaluation of our study in general. However, video-based error counting of high-frequency mapping sessions was not significantly more difficult than low-frequency examinations, and dysarthria was not a severe problem for data analysis among our study cohort. In addition, no volunteer requested a reduction of stimulation frequency due to intolerable discomfort or pain. Therefore, it is clear that rTMS with frequencies up to 20 Hz, as used in this study, turned out to be safe and tolerable for the individual subject.

*P* values comparing optimal stimulation frequencies for Broca’s *vs*. Wernicke’s rTMS language mapping indicate no significance (Table 2), and this shows that there is no specific correlation between the optimal frequencies of these two areas in our study. Therefore, at least in our study, the stimulation frequency determined for one of these spots is not likely to be able to predict the optimal frequency for the other one. This leads to the assumption that stimulation frequencies for rTMS language mapping should be determined separately for Broca’s and Wernicke’s areas.

In summation, as mentioned before, a higher stimulation frequency is correlated with a higher number of naming errors within Broca’s area and also within Wernicke’s area in our study. This finding indicates that high-frequency rTMS is needed to successfully disrupt linguistic functions, whereas low-frequency rTMS is not able to interfere with this on the same level [33]. Therefore, we come to the conclusion that language-related cortical areas are more prone to errors when stimulated with higher frequencies.

### Stimulation intensity

In keeping with our findings concerning stimulation frequencies, higher stimulation intensity also led to higher numbers of language disturbances than lower intensities (Tables 2 and 5). Epstein *et al*., for example, started with a stimulation intensity of 120% RMT and increased it, if necessary, up to 150% RMT [17]. The ranges of their and our applied intensities have only 120% RMT in common, which leads to the highest error number in our study. Epstein *et al*. did not systematically examine the single impact of stimulation intensities on human language function in their study, but it featured a positive correlation of intensity and discomfort [17], which is congruent with our findings. In Pascual-Leone *et al*.’s study, rTMS induced more reproducible naming errors when performed with high stimulation intensities [39]. Moreover, in Epstein *et al*., the authors described the occurrence of complete speech arrest, on average, at 116% RMT (range: 100% to 137% RMT) and stated that stronger magnetic stimulation is likely to produce more prominent effects on language functions [43], which are compatible with our stimulation results.

Similar to the *P* values comparing optimal coil angulations and stimulation frequencies, *P* values for the comparison of optimal intensities (Broca *vs*. Wernicke) indicate no statistically significant difference (Table 2). Therefore, we suggest that rTMS stimulation intensities should also be determined separately for Broca’s and Wernicke’s areas to optimize the impacts of rTMS on language-related brain areas. Mainly due to the relatively small size of the examined cohort, this recommendation has to be confirmed by future studies on rTMS language mapping.

As a consequence of our present study and literature review, the ranges for optimal stimulation intensities do not seem to be as controversial as those for coil angulations or for stimulation frequencies. Therefore, we can conclude that good rTMS language mapping results can already be achieved regularly with 100% RMT in most of individuals, but results can be improved significantly when higher intensities are used. This can be due to the fact that low stimulation intensities are able to interfere with language-related neuronal networks for only a short amount of time or to even enhance language functions, whereas high-intensity rTMS is likely to evoke a longer lasting effect on language functions, which results in an increased language impairment [33].

### Selection of stimulation targets

Previous language mapping approaches and personal experience have shown that left-hemispheric Broca’s area and Wernicke’s area are especially prone to naming errors during rTMS [24,25,31], which qualified both regions as appropriate stimulation targets for assessing the impact of rTMS intensity, frequency, and coil angulation on language performance during an object naming paradigm. Although rTMS to these cortical regions has routinely led to comparatively high error rates when compared with other stimulation spots, we have to be aware of the fact that the impact of the parameters evaluated in the present study might potentially be different with respect to other areas. Therefore, further studies investigating more distributed language-related areas might be helpful.

Moreover, the terms ‘Broca’s area’ and ‘Wernicke’s area’ are used to describe the spatio-anatomical cortical areas that were stimulated in the present study. In this context, we have to be aware of the modern literature favoring a widespread network responsible for language production and comprehension, which includes, but clearly exceeds the regions of Broca’s and Wernicke’s areas [44,45]. As aforementioned, the selection of the study’s stimulation targets is predominantly based on practical rTMS language mapping experience; therefore, any investigation of actual language organization models using rTMS is out of the scope of the present study.

### Safety aspects of rTMS

Although no adverse events were observed in the present study, we have to be aware of the principal risk of undesirable effects in the course of rTMS [46-48]. Concerning possible side effects, Wassermann mainly reports on epileptic seizures but also on hearing loss or effects on mood and cognition, for example [48]. Indeed, early TMS-based studies reported on the induction of seizures within a minority of patients [49,50], but none of the TMS-induced seizures described in previous publications led to permanent physical sequelae. More important, recent rTMS language trials of our and other groups did not lead to any seizures or other adverse events (except temporary headache) in patients or healthy volunteers [19,21,23,28-30].

Although several rTMS language mapping studies were published over the last years, distinct evidence-based data concerning limitations of stimulation intensity, stimulation frequency, and the number of applied stimulation pulses are still lacking regarding language mapping in particular. However, due to the fact that rTMS-based language mapping is usually performed with 80% to 120% RMT and a frequency of ≤20 Hz [19,21,23,28-30], the currently available safety limits should remain unaffected [47,48].

## Conclusions

Coil angulation, stimulation frequency, and stimulation intensity have a significant impact on language performance during rTMS language mapping. Variation of only one of these parameters already leads to a clearer impairment of language performance. Therefore, individually adapted stimulation parameters must be determined prior to language mapping in order to improve mapping results for each subject. Moreover, even high stimulation frequencies and intensities are safe and tolerable, at least in this small series.

## Abbreviations

Ap: anterior-posterior
APB: abductor pollicis brevis muscle
DCS: direct cortical stimulation
ECoG: electrocorticography
EHI: Edinburgh Handedness Inventory
EMG: electromyography
Hz: hertz
IPI: inter-picture interval
MEP: motor evoked potentials
MRI: magnetic resonance imaging
TMS: transcranial magnetic stimulation
nTMS: navigated transcranial magnetic stimulation
RMT: resting motor threshold
rTMS: repetitive navigated transcranial magnetic stimulation
SD: standard deviation
VAS: visual analog scale
