# Erratum to: The impact of repetitive navigated transcranial magnetic stimulation coil positioning and stimulation parameters on human language function

**DOI:** 10.1186/s40001-015-0155-z

**Published:** 2015-09-02

**Authors:** Nico Sollmann, Sebastian Ille, Thomas Obermueller, Chiara Negwer, Florian Ringel, Bernhard Meyer, Sandro M Krieg

**Affiliations:** Department of Neurosurgery, Klinikum rechts der Isar, Technische Universität München, Ismaninger Str. 22, 81675 Munich, Germany; TUM-Neuroimaging Center, Klinikum rechts der Isar, Technische Universität München, Ismaninger Str. 22, 81675 Munich, Germany

## Erratum to: European Journal of Medical Research (2015) 20:47 DOI 10.1186/s40001-015-0138-0

Following publication of our article [1], it was noticed that an error to the presentation of Table 2 was introduced by the publisher. The corrected table is given below (Table 1) and has been updated in the original publication [1].

**Table 1:** Summary of stimulation parameters and coil angulations

Subject		Broca					Wernicke					p
		#1	#2	#3	#4	#5	#1	#2	#3	#4	#5	–
Optimal coil angulation (in °)	To ap orientation	225	90	90	135	90	90	45	180	270	135	0.8288
	To reference gyrus	135	0	0	45	0	0	315	90	180	45	0.1945
Optimal stimulation frequency (in Hz)		10	10	20	20	7	20	20	20	7	10	0.7337
Optimal stimulation intensity (% RMT)		100	120	120	120	100	120	100	100	120	120	0.9025
Electrical field strength (in V/m)		85	120	95	100	70	95	75	105	90	75	0.7526
